# Complicaciones tromboembólicas asociadas con tuberculosis: reporte de un caso pediátrico

**DOI:** 10.7705/biomedica.5195

**Published:** 2020-12-09

**Authors:** Natalia Osorio, Martha Mónica Reyes

**Affiliations:** 1 Facultad de Medicina, Universidad CES, Medellín, Colombia Universidad CES Facultad de Medicina Universidad CES Medellín Colombia

**Keywords:** tuberculosis, embolia pulmonar, trombosis de la vena, antituberculosos, anticoagulantes, adolescente, Tuberculosis, pulmonary embolism, venous thrombosis, antituberculosis drugs, anticoagulants, adolescent

## Abstract

La tuberculosis es una de las enfermedades infecciosas más comunes en el mundo. Aunque la mortalidad en niños es prácticamente nula cuando el diagnóstico y el tratamiento son oportunos, puede asociarse con complicaciones como la trombosis venosa profunda y la superficial a partir de la respuesta inflamatoria sistémica frente a la infección, lo que propicia la coagulación y ocasiona una significativa morbimortalidad.

Se reporta el caso de una adolescente de 14 años con tuberculosis pulmonar en tratamiento combinado quien, de forma atípica, presentó dos episodios de tromboembolia venosa: el primero en el riñón y el segundo en los pulmones. Tras descartar el síndrome nefrótico y el antifosfolipídico, los estudios de tomografía de tórax y abdomen fueron una herramienta fundamental para su diagnóstico. Se inició tratamiento con heparina de bajo peso molecular con mejoría de los síntomas. Teniendo en cuenta las necesidades de anticoagulación no fue posible realizar estudios adicionales de ampliación.

Las complicaciones tromboembólicas en pacientes con tuberculosis y sin otros factores de riesgo obligan a considerar el efecto coagulante que resulta de la reacción inflamatoria sistémica, la cual podría, por sí sola, ser la causa de una complicación significativa pero prevenible, aunque frecuentemente escapa al diagnóstico. En este sentido, se recomienda considerar la posibilidad de la tromboembolia venosa en estos pacientes y hacer un seguimiento estricto que permita aplicar el tratamiento anticoagulante tempranamente y prevenir, así, resultados adversos.

## Abstract (en)

Tuberculosis is one of the most common infectious diseases around the world. With timely diagnosis and treatment, mortality in children is practically zero. It is usually associated with a diverse number of complications that can cause significant morbidity and mortality, such as deep and superficial vein thrombosis. This event has been associated with a procoagulant state caused by the systemic inflammatory response to infection.

We report the case of a 14-year-old adolescent with pulmonary tuberculosis under the initial four-drug regimen. She presented two episodes of venous thromboembolism, the first in the kidneys and the second in the lungs. After ruling out diseases such as nephrotic and antiphospholipid antibody syndrome, chest and abdomen tomographies were performed as a fundamental tool for the diagnosis. Thereafter, treatment with low molecular weight heparin was initiated and the symptoms improved. Given the requirement for anticoagulation, further image studies could not be done.

Thromboembolic complications in patients with no other risk factors, associated only with a previous pulmonary tuberculosis diagnosis, offer evidence to consider the procoagulant effect resulting from the systemic inflammatory response that, by itself, could be the cause of a serious complication, often underdiagnosed but also preventable. Therefore, it is recommended to consider the predisposition for venous thromboembolism in these patients and to establish strict surveillance so early anticoagulant therapy can be provided to prevent adverse outcomes.

La tuberculosis es una de las enfermedades infecciosas más comunes y una de las principales causas de mortalidad globalmente. Afecta a un tercio de la población mundial, de la cual el 11 % corresponde a niños, entre quienes se registra hasta un millón de casos nuevos cada año ^1^^,^^2^. Además, la tuberculosis predomina en poblaciones con barreras de acceso a los servicios de salud y constituye un reto diagnóstico en el caso de los niños ya que, muchas veces, solo tras la búsqueda activa de la fuente de contacto en los adultos, se logra esclarecer el diagnóstico en ellos ^1^.

Asimismo, la tuberculosis se asocia con diversas complicaciones que implican una morbimortalidad significativa. Entre dichas complicaciones se han descrito la trombosis venosa profunda y la superficial hasta en el 24 % de los diagnósticos *post mortem*^3^; se estima que la incidencia fluctúa entre el 3 y el 4 % de los casos de tuberculosis, principalmente en los de la forma miliar, lo que evidencia que el riesgo aumenta según la gravedad de la enfermedad ^4^^-^^6^.

Este tipo de complicación se asocia con estados propiciadores de la inflamación y la coagulación ocasionados por la respuesta inflamatoria sistémica frente a la infección y la presencia de anticuerpos antifosfolípidos, especialmente los anticuerpos antimicroglobulina beta-2 y, más recientemente, los anticuerpos antifosfatidiletanolamina, así como de otras condiciones concomitantes como la compresión extrínseca por adenopatías propia del proceso de tuberculosis, o el reposo prolongado durante la hospitalización ^6^^-^^8^.

## Presentación de caso

Se presenta el caso de una paciente de 14 años con diagnóstico de tuberculosis de origen pulmonar y sin antecedentes patológicos previos. El diagnóstico se confirmó en baciloscopias seriadas positivas y recibió el tratamiento compuesto con isoniacida, rifampicina, pirazinamida y etambutol. Un mes después de iniciar la medicación, consultó por dolor localizado en la fosa renal izquierda, hematuria macroscópica y disuria. En una tomografía de abdomen con contraste se observaron cambios inflamatorios en el riñón izquierdo, y un trombo en la vena renal izquierda con extensión dentro del riñón y la vena cava inferior. Se describieron, asimismo, edema y captación del contraste en el urotelio del costado izquierdo, principalmente el tercio proximal, secundario a ureteritis de probable origen tuberculoso.

Ante los hallazgos descritos, se inició el tratamiento con heparina de bajo peso molecular y se hicieron los estudios en búsqueda de trombofilia; se encontró prolongación del tiempo parcial de tromboplastina y aumento. Sin embargo, dado que la paciente estaba recibiendo anticoagulante y existía el riesgo de falsos negativos, no fue posible hacer otros exámenes paraclínicos. En este punto se decidió darle de alta y continuar con el seguimiento ambulatorio.

Cuatro meses después, la paciente consultó de nuevo con disnea, tos no productiva, dolor torácico y aumento de la necesidad de oxígeno. Durante la anamnesis, refirió haber suspendido por decisión propia el tratamiento con heparina de bajo peso molecular. En el examen físico se encontró taquicardia, taquipnea e hipoxemia, por lo que se la hospitalizó.

Durante la hospitalización, se practicaron baciloscopias seriadas de control, las cuales fueron negativas; además, se descartaron enfermedades comúnmente asociadas con la trombosis, como el síndrome nefrótico, pues la concentración de albúmina era normal; el síndrome antifosfolipídico, pues no se demostraron anticuerpos anticardiolipina ni antiglucoproteína beta 2 (cuadro 1), y el lupus eritematoso sistémico, pues los anticuerpos anti-ADN y anti-Smith fueron negativos. No obstante, en otros exámenes de laboratorio se encontraron niveles de dímero D anormalmente elevados, lo cual era indicativo de tromboembolia pulmonar.

**Cuadro 1:** Exámenes paraclínicos practicados a la paciente

Laboratorio	Resultado	Valores de referencia
Cardiolipina, anticuerpos IGG (GPL)	2,63	<10 negativo
Cardiolipina, anticuerpos IGM (MPL)	5,13	<7 negativo
Tiempo de tromboplastina (s)	43,3	23,9-34,9
Anticoagulante lúpico (s)	47,0	30,4-45,3
Proteína S de coagulación (%)	64,8	55-123
Proteína S, antígeno total (%)	122	70-140
Proteína C de coagulación (%)	105,5	70-140
Dímero D (µg FEU/ml)	6,75	0-0,49
Glucoproteína beta 2, anticuerpos IGG (SGU)	1,45	0-20
Anticuerpos antinucleares	Positivo 1:80 patrón moteado	Negativo
Anticuerpos anti-Ro	0,1	0-0,9
Anticuerpos anti-La	0,17	0-0,9
Anticuerpos anti-Smith	0,12	0-0,9
Anticuerpos anti-Sm/RNP	0,10	0-0,9
Anticuerpos anti-ADN	Negativo	Negativo
Albúmina (g/dl)	3,5	3,4-5,4

**Figura 1:**
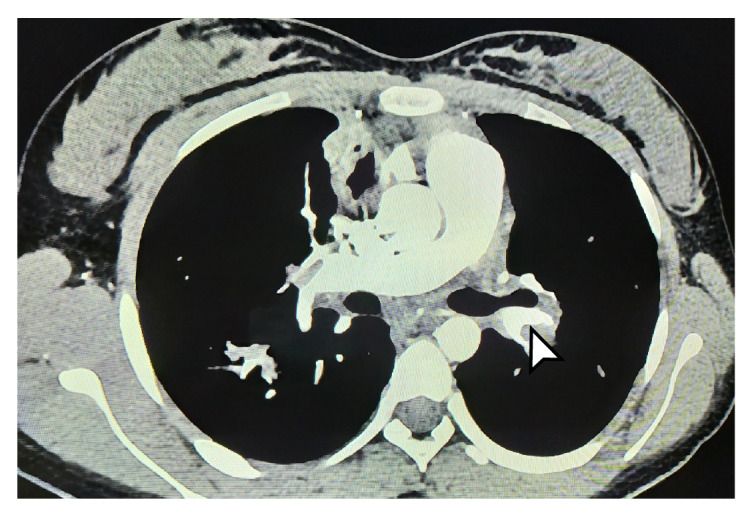
Tomografía computarizada de tórax. La flecha blanca señala

Los estudios se complementaron con tomografías de tórax y de abdomen con contraste, las cuales evidenciaron tromboembolia pulmonar grave (figuras 1 y 2) con múltiples trombos bilaterales, áreas pequeñas de infartos pulmonares, nódulos pulmonares múltiples, y embolia venosa renal bilateral con trombo que comprometía la vena cava superior. Se decidió reiniciar el tratamiento anticoagulante con heparina de bajo peso molecular, con lo cual los síntomas respiratorios mejoraron, lo que permitió dar de alta a la paciente y retomar el seguimiento por consulta externa.

**Figura 2:**
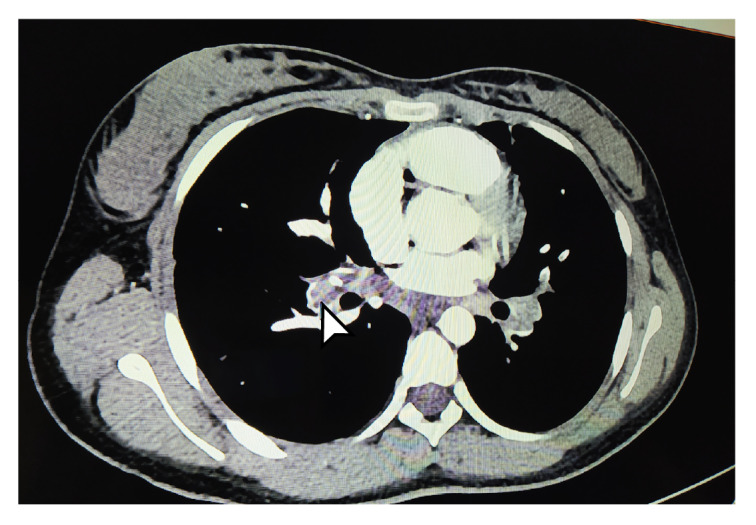
Tomografía computarizada de tórax La flecha indica trombosis un gran trombo en la vena pulmonar izquierda. extensa de las venas pulmonares (derecha e izquierda).

### Consideraciones éticas

Según el Artículo 11 de la Resolución número 008430 del 4 de octubre de 1993, esta investigación cumple con las condiciones establecidas por el Ministerio de Salud de la República de Colombia para ser clasificada como una investigación sin riesgo, ya que solo implicó la revisión de la historia clínica para el reporte de caso. El estudio fue aprobado por el comité de ética de la institución donde fue atendida la paciente y el guardián legal de la paciente autorizó la publicación mediante un consentimiento informado.

## Discusión

El estado propicio para la coagulación secundaria a la tuberculosis se produce principalmente en las fases tempranas de la enfermedad, sin embargo, también se han descrito casos tardíos ^9^. Es un proceso multifactorial, es decir, la respuesta inflamatoria inherente al proceso infeccioso subyacente compromete los niveles de antitrombina III y proteína C, y produce un importante aumento del fibrinógeno sérico y del dímero D, así como daño directo del endotelio con trombocitosis reactiva, lo que favorece el proceso de agregación plaquetaria y la trombosis ^8^, y se refleja en la prolongación de los tiempos de coagulación.

Por otro lado, se ha establecido que el tratamiento compuesto de cuatro medicamentos antituberculosos es un factor de riesgo de trombosis, especialmente por la rifampicina. Este fármaco se ha asociado con proliferación del retículo endoplásmico liso en las células hepáticas; además, al ser de metabolismo hepático, causa la inducción de la enzima citocromo P450, lo que compromete la producción de las enzimas anticoagulantes y favorece su rápida depuración, lo cual altera la homeostasis a favor de un estado propicio a la coagulación ^10^^-^^12^.

Otro hallazgo frecuente es la presencia de anticuerpos antifosfolípidos como el anticoagulante lúpico y los anticuerpos antifosfatidiletanolamina. El primero se ha relacionado con la deficiencia parcial de proteína S, un factor anticoagulante conocido que, al disminuir, promueve la tendencia coagulante, en tanto que los segundos aumentan como reacción al proceso infeccioso de la tuberculosis ^4^^,^^13^^,^^14^. En el lupus eritematoso sistémico y el síndrome de anticuerpos antifosfolípidos, se ha establecido su papel como factor protrombótico dado que la fosfatidiletanolamina desempeña una función anticoagulante ^7^.

Otros factores que propician la coagulación son las adenomegalias que, por acción local, pueden ocluir vasos e interrumpir el flujo laminar ^15^, y aquellos que producen estasis vascular, como el reposo prolongado en pacientes hospitalizados durante periodos extensos, lo cual limita la movilidad y propicia la aparición de la tríada de Virchow ^4^.

Entre las principales complicaciones tromboembólicas, se ha descrito el compromiso de las venas de miembros inferiores, las hepáticas y los senos venosos cerebrales ^13^^,^^16^. En el presente caso, hubo daño en la vena renal con extensión significativa hasta la vena cava inferior y la superior.

El tratamiento para las complicaciones trombóticas son los anticoagulantes. En pediatría se utilizan tradicionalmente tres tipos: la heparina no fraccionada, la heparina de bajo peso molecular y los antagonistas de la vitamina K. El primero es un medicamento que, al unirse a la antitrombina III, potencia la actividad inhibitoria de la coagulación, posee una vida media corta, su acción es de inicio rápido y se dispone de antídoto. Se usa sobre todo para la profilaxis en procedimientos y la permeabilidad de accesos vasculares en bolos de 75 a 100 unidades/kg, con dosis de mantenimiento que dependen de la edad: en menores de dos meses, aproximadamente de 28 unidades/kg por hora, y en niños mayores de esta edad, de 18 a 20 unidades/kg por hora ^17^^,^^18^.

Por otra parte, la heparina de bajo peso molecular actúa inhibiendo el factor Xa mediante la activación de la antitrombina III. Entre sus ventajas, resalta la vida media prolongada y el bajo riesgo de interacción farmacológica. El rango terapéutico se infiere de los resultados en población adulta y se basa en el seguimiento de los niveles del antifactor Xa en suero. En neonatos, la dosis media para alcanzar el rango terapéutico varía entre 1,6 y 2 mg/kg dos veces al día y, en niños mayores, entre 1,12 y 1,9 mg/kg dos veces al día ^17^^,^^18^.

Por último, la acción de los antagonistas de la vitamina K, que inhiben factores dependientes de la vitamina como el X, IX, VII, II y las proteínas C y S, varía según la dieta y los medicamentos que ingiera el paciente. Por ejemplo, los niños alimentados con fórmulas infantiles pueden ser resistentes al medicamento porque dichas fórmulas vienen enriquecidas con esta vitamina, por lo que la supervisión es difícil y requiere vigilancia estrecha. La dosis de inicio es de 0,1 a 0,2 mg/kg con rangos terapéuticos del índice internacional normalizado *(International Normalized Ratio,* INR) entre 2 y 3. En la población pediátrica, especialmente en preescolares y lactantes, la presentación es una limitación, puesto que está disponible únicamente en tabletas y su recomposición en formulación líquida no es segura ^17^^,^^18^.

Cabe aclarar que, además de los ya mencionados, los anticoagulantes de nueva generación se han probado en la población pediátrica y están indicados en la trombocitopenia inducida por heparina. Actualmente, se encuentran disponibles los inhibidores de trombina (bivalirudina y argatroban), cuya adecuada seguridad ya ha sido documentada, así como pocos episodios de sangrado y la resolución rápida de los coágulos. El fondaparinux, por su parte, actúa inhibiendo el factor Xa y tiene un perfil de seguridad aceptable, requiere una dosis única al día y se supervisa midiendo los niveles del factor anti-Xa; se recomienda una dosis de 0,1 mg/kg por día y su rango terapéutico es de 0,5 a 1 mg/L ^17^^,^^18^.

En el presente caso, se llegó a considerar el empleo de warfarina, pero se sabe que los efectos de los cumarínicos se ven atenuados por el uso concomitante de rifampicina y el metabolismo hepático ya descrito ^4^^,^^10^^,^^11^, lo que supone una mayor dificultad para mantener el INR aceptado ^19^. Por ello, la alternativa recomendada fue la heparina de bajo peso molecular, la cual se utilizó tras el diagnóstico de trombosis.

En algunos estudios se ha propuesto el manejo con heparina de bajo peso molecular como profilaxis ^4^^,^^10^^,^^20^, sin embargo, no hay un consenso establecido sobre su inicio. Se plantea que, en casos graves de tuberculosis pulmonar o miliar, se debería iniciar la anticoagulación simultáneamente con el tratamiento compuesto, ya que las alteraciones hematológicas secundarias de la enfermedad usualmente se resuelven tras un mes de tratamiento antituberculoso ^4^^,^^20^.

No se ha establecido el tiempo mínimo de tratamiento con anticoagulación. En la revisión bibliográfica se encontraron reportes de casos de pacientes tratados durante tres meses que posteriormente hubo que extender hasta seis meses ^20^. Monagle, *et al.,* publicaron en el 2012 una guía para el tratamiento antitrombótico en neonatos y niños, recomendando el uso de heparina no fraccionada o heparina de bajo peso molecular en quienes presenten trombosis unilateral de la vena renal con extensión a la vena cava superior de una duración aproximada de tres semanas hasta seis meses, como en el presente caso ^21^. También, se encontraron publicaciones sobre el uso de antagonistas de la vitamina K. Sangani, *et al.,* por ejemplo, reportaron el caso de una paciente de 11 años con tuberculosis y trombosis en la vena cava inferior y en la femoral, tratada con warfarina durante seis meses ^22^.

Es claro que se debe instaurar de forma temprana el tratamiento compuesto con los cuatro fármacos antituberculosos y mantenerse atento a los signos que sugieran complicaciones secundarias al proceso propiciador de la coagulación para iniciar oportunamente el tratamiento anticoagulante, incluso en aquellos pacientes sin factores de riesgo adicionales.

## Conclusiones

La tuberculosis sigue siendo una causa importante de morbimortalidad en Colombia y es importante documentar las complicaciones secundarias y su frecuencia en la población pediátrica, con el fin de recopilar los datos en cohortes más grandes e informar el comportamiento de las variables hematológicas en este grupo etario. El tratamiento óptimo de las complicaciones tromboembólicas se basa en la vigilancia médica y en la sospecha diagnóstica ante hallazgos clínicos o paraclínicos sugestivos, por lo cual se recomienda un seguimiento estricto de estos pacientes.
